# Global risk factor rankings: the importance of age-based health loss inequities caused by alcohol and other risk factors

**DOI:** 10.1186/s13104-015-1207-8

**Published:** 2015-06-09

**Authors:** Kevin D Shield, Jürgen Rehm

**Affiliations:** Centre for Addiction and Mental Health (CAMH), Toronto, Canada; Institute of Medical Science, University of Toronto, Toronto, Canada; PAHO/WHO Collaborating Centre for Mental Health and Addiction, 33 Russell Street, Toronto, ON M5S 2S1 Canada; Dalla Lana School of Public Health (DLSPH), University of Toronto, Toronto, Canada; Institute of Clinical Psychology and Psychotherapy, Technische Universität, Dresden, Germany; Department of Psychiatry, University of Toronto, Toronto, Canada

**Keywords:** Health equity, Global Burden of Disease, Disability Adjusted Life Years, Risk factor, Alcohol

## Abstract

**Background:**

Achieving health equity is a priority of the World Health Organization; however, there is a scant amount of literature on this topic. As the underlying influences that determine health loss caused by risk factors are age-dependent, the aim of this paper is to examine how the risk factor rankings for health loss differ by age.

**Methods:**

Rankings were based on data obtained from the 2010 Global Burden of Disease study. Health loss (as measured by Disability Adjusted Life Years lost) by risk factor was estimated using Population-Attributable Fractions, years of life lost due to premature mortality, and years lived with disability, which were calculated for 187 countries, 20 age groups and both sexes. Uncertainties of the risk factor rankings were estimated using 1,000 simulations taken from posterior distributions

**Results:**

The top risk factors by age were: household air pollution for neonates 0–6 days of age [95% uncertainty interval (UI): 1 to 1]; suboptimal breast feeding for children 7–27 days of age (95% UI: 1–1); childhood underweight for children 28 days to less than 1 year of age and 1–4 years of age (95% UI: 1–2 and 1–1, respectively); iron deficiency for children and youth 5–14 years of age (95% UI: 1–1); alcohol use for people 15–49 years of age (95% UI: 1–2); and dietary risks for people 50 years of age and older (95% UI: 1–1). Rankings of risk factors varied by sex among the older age groups. Alcohol and smoking were the most important risk factors among men 15 years of age and older, and high body mass and intimate partner violence were some of the most important risk factors among women 15 years of age and older.

**Conclusions:**

Our analyses confirm that the relative importance of risk factors is age-dependent. Therefore, preventing harms caused by various modifiable risk factors using interventions that target people of different ages should be a priority, especially since easily implemented and cost-effective public health interventions exist.

**Electronic supplementary material:**

The online version of this article (doi:10.1186/s13104-015-1207-8) contains supplementary material, which is available to authorized users.

## Background

Achieving health equity is a top priority of the World Health Organization (WHO), as achieving health targets without an equitable distribution is of limited value [1]; however, there is a scant amount of literature on global health equity. Accordingly, the WHO has prioritized identifying and quantifying the determinants of health inequities [1, 2], and, thus, an investigation of the distribution of health loss caused by risk factors among sub-populations can increase our understanding of how health inequities arise and how best they should be addressed [3–5]. The 2010 Comparative Risk Assessment (CRA) publication ranked risk factors by health loss [measured as Disability Adjusted Years of Life (DALYs) lost] [6]; however, as the underlying influences that determine health loss caused by risk factors are age-dependent (namely, exposure, and individual vulnerability to, and health outcomes from, causally-related diseases and injuries), the rankings of risk factors are hypothesized to depend on age. Accordingly, we examined the rankings of risk factors based on the DALYs lost by age based on the 2010 CRA findings.

## Methods

Data were obtained from the 2010 Global Burden of Disease (GBD) study, for which the authors of this article played a role in the estimation of the burden of disease attributable to alcohol consumption (see [7] for a general overview of the methods used in the 2010 GBD study). The 2010 GBD CRA study estimated the number of DALYs lost by risk factor by calculating the number of years of life lost (YLL) due to premature mortality (see [8, 9] for more information on mortality data collection and estimation) and the number of years lived with disability (YLD) (see [10, 11] for more information on morbidity data collection and estimation), and also calculated the mortality and morbidity Population-Attributable Fractions (PAFs) by risk factor for 187 countries, 20 age groups and both sexes. PAFs represent the burden of a disease (either mortality or morbidity) that would not be present under a counterfactual scenario of everyone being exposed to the theoretical-minimum-risk exposure distribution (the amount of exposure that leads to the lowest burden of disease). For alcohol and tobacco in particular, this theoretical-minimum-risk exposure distribution assumed that every person abstained from alcohol and tobacco for their entire life, while for risk factors such as diet and blood pressure, the theoretical-minimum-risk exposure distribution assumed the healthiest blood pressure and dietary intake. For each risk factor, PAFs for mortality and morbidity were calculated using prevalence of exposure status and exposure distributions for people who were exposed to a risk factor (see [6] for information on data sources of the exposure status) and using the relative risks (RR) for each disease condition and injury that was deemed to be causally related to the risk factor (see [6] for a list of diseases, conditions and injuries causally related to each of the risk factors examined). The mortality and morbidity risk factor PAFs were then applied to the number of YLL and YLD respectively, with the resulting YLL and YLD being summed to estimate the DALYs lost attributable to each risk factor.

To estimate the uncertainty of the risk factor rankings, simulation analyses were employed using 1,000 draws from the posterior distribution of exposure, RRs, YLL and YLD for each age, sex and country. Simulations accounted for the correlation of uncertainty between measures. The 2.5 and 97.5 percentiles of the simulations were used as an indicator of the 95% uncertainty intervals (UI).

### Ethics

As this was an analysis of secondary data, no ethics approval from the Centre for Addiction and Mental Health (the authors’ main research institution) was required.

## Results

**Figure 1:**
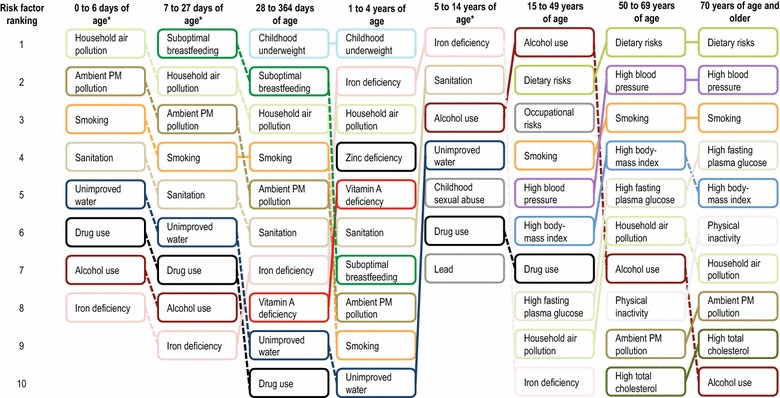
Global risk factor rankings (top 10) for the total burden of disease (measured in Disability Adjusted Life Years lost) by age in 2010. *Asterisks* data for exposure and risk relationships for all risk factors are not available for these groups, and thus fewer than ten risk factors are presented

The top risk factors by age were: insufficient weight for children 5 years of age and younger (95% UI: 1–1); iron deficiency for children and youth 5–14 years of age (95% UI: 1–1); alcohol use for people 15–49 years of age (95% UI: 1–2); and dietary risks for people 50 years of age and older (95% UI: 1–2). In addition, smoking and high blood pressure had a greater impact on people in older age groups. As indicated above, alcohol (the fifth leading cause of health loss (95% UI: 4–7)) had the greatest effect on people in young adulthood up to 49 years of age. Figure 1 outlines the risk factor rankings by age in 2010.

Rankings of risk factors varied by sex for older age groups only. The risk factor rankings for children and youth (14 years of age and younger) showed little variation by sex. Among older age groups, alcohol, smoking and occupational risks were more important risk factors among men 15 years of age and older and high body mass index and intimate partner violence were more important risk factors among women 15 years of age and older. See Additional file 1: Figure A1 and Additional file 2: Figure A2 for the risk factor rankings in 2010 by age for women and men respectively.

## Discussion

Our analysis confirms that the relative importance of risk factors is age-dependent. The findings concerning the top five risk factors, namely dietary risks, high blood pressure, smoking, household air pollution, and alcohol are especially important as a large variance in the risk factor rankings by age was observed. These differences in risk factor rankings by age should be taken into account, especially as utilitarian ageism is often observed in medical practice which leads to the prioritization of treatments for health loss among the young (as the old have lived longer) [12, 13].

Furthermore, the risk factor rankings for children and youth (14 years of age and younger) showed little variation by gender, while the risk factors for people 15 years of age and older showed a large variation by gender. This is likely due to risk exposure not varying by gender for children and youth (14 years of age and younger) when compared to the risk exposure differences by gender for people 15 years of age and older [14]. In particular, smoking and alcohol—two risk factors more prevalent among men than among women 15 years of age and older [15, 16]—caused a relatively larger burden of disease when compared to other risk factors for men when compared to women. Intimate partner violence was a more important risk factor among women 15 years of age and older, as women are much more likely to be the target of intimate partner violence [17]. Lastly, high body mass index affected women more than other risk factors when compared to men; other risk factors such as smoking and alcohol caused a smaller relative burden of disease among women.

It should be noted that there were some limitations respecting the estimation of the burden attributable to alcohol consumption in the 2010 GBD study. Specifically, the burden attributable to alcohol consumption is likely underestimated, as conditions such as alcohol abuse and HIV/AIDS were not included in these calculations, and neither were any mental health conditions other than alcohol dependence (for a more recent estimate including these conditions see [18]). Furthermore, with respect to risk factors for neonates 0–6 days of age, children 6–27 days of age and children and youth 5–14 years of age, data were missing on the exposure to certain risk factors and risk relationships, and, therefore, calculation of the effects of these risk factors was impossible. For instance, the effects of smoking on children, youth and young adults aged 5–25 years was set to zero, even though tobacco smoking is likely to cause some burden of disease among people in this age group.

Health inequities should also be considered in light of the feasibility and effectiveness of interventions. As recommended by the WHO, effective interventions are replicable, sustainable, scalable, and politically, economically and technically feasible [1]. Of the top risk factors, alcohol and smoking interventions rank high on these dimensions. Lastly, several alcohol and smoking interventions were designated as “best buys” by the WHO as they are more cost-effective than most other interventions designed for other risk factors [19]; even the more cost-effective interventions for dietary risks, high blood pressure, and household air pollution are costly when compared to the most cost-effective interventions for alcohol and tobacco.

Furthermore, public health policy measures aimed at smoking and alcohol, such as advertising restrictions, are more prevalent for smoking than similar public health policy measures aimed at alcohol consumption. (This may be due, in part, to alcohol having a protective effect on ischemic cardiovascular diseases and diabetes at low consumption amounts [20]). Additionally, the fact that alcohol use is the top risk factor among people 15–49 years of age is important, as investment in preventing mortality from injury has fallen behind other causes of death, such as HIV/AIDS and reproductive health [21], and mental health concerns have been overlooked in terms of public health programming, especially in young people [22] where injuries and neuropsychiatric conditions are greatly impacted by alcohol consumption [23].

## Conclusion

Although the risk factor rankings by age are important for equity considerations, analyses are needed to determine the underlying causes of health loss by dimensions other than age, such as by income, education, and race/ethnicity [24].

## Additional files

Additional file 1:
** Figure A1.** Global risk factor rankings (top 10) for the total burden of disease (measured in Disability Adjusted Life Years lost) by age for women in 2010. Additional file 2:
** Figure A2.** Global risk factor rankings (top 10) for the total burden of disease (measured in Disability Adjusted Life Years lost) by age for men in 2010.

## Abbreviations

CRA: Comparative Risk Assessment
DALYs: Disability Adjusted Years of Life
GBD: Global Burden of Disease
PAFs: Population-Attributable Fractions
RR: relative risks
UI: uncertainty intervals
WHO: World Health Organization
YLD: years lived with disability
YLL: years of life lost
