# Supplementary material for: Global risk factor rankings: the importance of age-based health loss inequities caused by alcohol and other risk factors
Source: BMC Res Notes. 2015 Jun 9;8:231. doi: 10.1186/s13104-015-1207-8 (PMC4467665; doi:10.1186/s13104-015-1207-8)
Supplement: Additional file 1: — Figure A1. Global risk factor rankings (top 10) for the total burden of disease (measured in Disability Adjusted Life Years lost) by age for women in 2010. [file 13104_2015_1207_MOESM1_ESM.pdf]

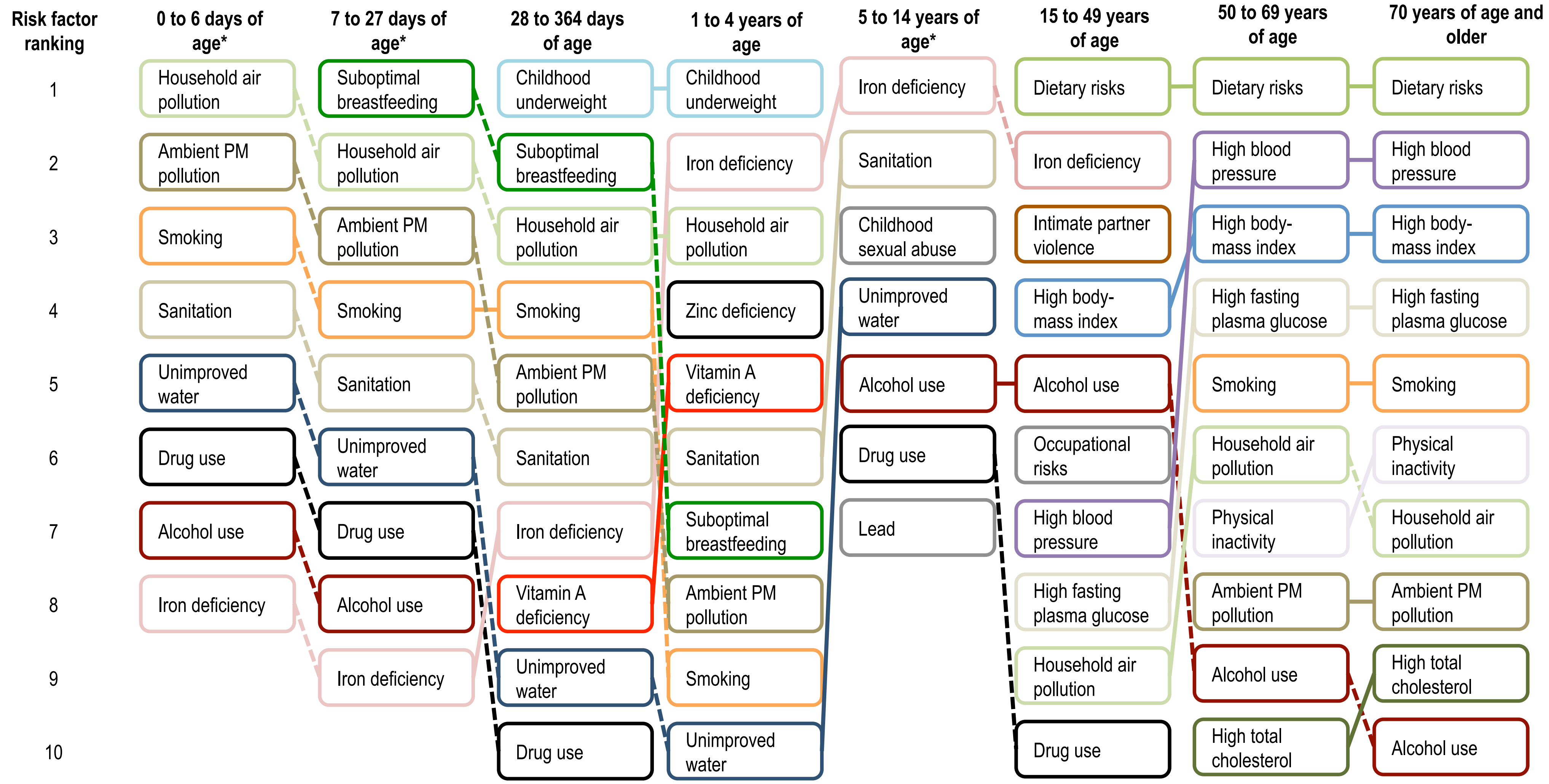

* Data for exposure and risk relationships for all risk factors are not available for these age groups, and thus fewer than 10 risk factors are presented

Figure A1. Global risk factor rankings (top 10) for the total burden of disease (measured in Disability Adjusted Life Years) by age for women in 2010
