# Supplementary material for: Global risk factor rankings: the importance of age-based health loss inequities caused by alcohol and other risk factors
Source: BMC Res Notes. 2015 Jun 9;8:231. doi: 10.1186/s13104-015-1207-8 (PMC4467665; doi:10.1186/s13104-015-1207-8)
Supplement: Additional file 2: — Figure A2. Global risk factor rankings (top 10) for the total burden of disease (measured in Disability Adjusted Life Years lost) by age for men in 2010. [file 13104_2015_1207_MOESM2_ESM.pdf]

Risk factor ranking	0 to 6 days of age*	7 to 27 days of age*	28 to 364 days of age	1 to 4 years of age	5 to 14 years of age*	15 to 49 years of age	50 to 69 years of age	70 years of age and older
1	Household air pollution	Suboptimal breastfeeding	Childhood underweight	Childhood underweight	Iron deficiency	Alcohol use	Dietary risks	Dietary risks
2	Ambient PM pollution	Household air pollution	Suboptimal breastfeeding	Household air pollution	Sanitation	Occupational risks	Smoking	High blood pressure
3	Smoking	Ambient PM pollution	Household air pollution	Iron deficiency	Alcohol use	Dietary risks	High blood pressure	Smoking
4	Sanitation	Smoking	Smoking	Zinc deficiency	Unimproved water	Smoking	High fasting plasma glucose	High fasting plasma glucose
5	Unimproved water	Sanitation	Ambient PM pollution	Vitamin A deficiency	Childhood sexual abuse	High blood pressure	Alcohol use	High body-mass index
6	Drug use	Unimproved water	Sanitation	Sanitation	Drug use	Drug use	High body-mass index	Ambient PM pollution
7	Alcohol use	Drug use	Iron deficiency	Ambient PM pollution	Lead	High body-mass index	Ambient PM pollution	Physical inactivity
8	Iron deficiency	Alcohol use	Unimproved water	Smoking		High fasting plasma glucose	Household air pollution	Household air pollution
9		Iron deficiency	Vitamin A deficiency	Suboptimal breastfeeding		Household air pollution	Physical inactivity	Alcohol use
10			Drug use	Unimproved water		Ambient PM pollution	Occupational risks	High total cholesterol

* Data for exposure and risk relationships for all risk factors are not available for these age groups, and thus fewer than 10 risk factors are presented

Figure A2. Global risk factor rankings (top 10) for the total burden of disease (measured in Disability Adjusted Life Years) by age for men in 2010
